# Educational programmes for frail older people, their families, carers and healthcare professionals: A systematic review

**DOI:** 10.1007/s00508-021-01900-4

**Published:** 2021-07-01

**Authors:** Rachel J. Viggars, Andrew Finney, Barnabas Panayiotou

**Affiliations:** 1 North Staffordshire General Practice Federation, 69–71 Stafford Street, ST1 1LS Hanley, UK; 2 School of Nursing & Midwifery, Keele University, Stoke-on-Trent, ST5 5BG Staffordshire, UK; 3 Postgraduate School of Medicine, Keele University, Stoke-on-Trent, ST5 5BG Staffordshire, UK

**Keywords:** Training, Relatives, Self-care, Exercise, Nutrition

## Abstract

**Background:**

More people are living with frailty and requiring additional health and support services. To improve their management, the “Frailty: Core Capability Framework” in the United Kingdom recommends frailty education for older individuals, their families, carers and health professionals. We performed a systematic review of specific educational programmes for these groups.

**Methods:**

Electronic databases were searched using dedicated search terms and inclusion criteria. To improve accuracy, two reviewers carried out the screening and selection of research papers. Information from included studies was collected using a tailored data extraction template, and quality appraisal tools were used to assess the rigour of the studies. The findings were analysed to identify key themes.

**Results:**

A total of 11 studies met the criteria and were included in the review. The study populations ranged from 12 to 603 and the research designs were heterogeneous (6 qualitative; 2 randomised controlled trials; 1 quasi-experimental; 1 mixed methods; 1 cross-sectional study). Whilst some methodological shortcomings were identified, all studies contributed valuable information. The results underwent narrative synthesis, which elucidated four thematic domains: (1) accessibility of educational programmes, (2) empowerment, (3) self-care, and (4) health promotion (especially exercise and nutrition).

**Conclusion:**

Educational programmes for older people, their carers and health professionals are important for effective frailty prevention and management. To be maximally beneficial, they should be easily accessible to all target populations and include empowerment, self-care and health promotion. Further research should explore the formulation of widely applicable, user-friendly programmes and delivery formats that can be tailored to different client groups.

## Key summary points

Aim: To perform a systematic review of educational programmes for frail older people, their families, carers and healthcare professionals.

Findings: Eleven publications met the inclusion criteria. Narrative synthesis elucidated four thematic domains, i.e. Accessibility to education; empowerment; self-care; health promotion.

Message: To be maximally beneficial, educational interventions should be accessible to all target groups and incorporate empowerment, self-care and health promotion.

## Introduction

As the population ages, more people are living with frailty and consequently the demands on health and social care systems are significantly greater [1]. Currently, around 50% of people who are over the age of 65 years are living with some level of frailty [2]. Frailty is linked to the ageing process and multimorbidity and it can vary over time. Even relatively minor transient changes in physical or mental well-being may have a major impact on the functional state and outcome of frail older people [3].

Frailty can be regarded as a long-term condition (LTC) because it shares similar characteristics. Just like other LCTs, such as diabetes, heart disease, arthritis, obstructive pulmonary disease, frailty is also chronic, progressive and can fluctuate. It is manageable but not curable and adversely affects quality of life [4]. Considering frailty as an LTC, and appreciating its impact on people and their families, are now components of the general practitioners’ (GPs) contract in the United Kingdom (UK) and this includes recommendations for frailty prevention and management [5].

It is known that patients with LTCs have better outcomes if they and their carers receive education around their conditions [6, 7]. For example, best evidence suggests that patients with type 2 diabetes should receive structured education regarding their condition and ongoing management [8]. The Dunhill Medical Trust further emphasizes the important role of educating patients, their families, carers and healthcare personnel in relation to older people’s health and social care [9]. Through education of affected individuals, their families and carers, self-management of LTCs including frailty should form an important component of a person’s overall care plan [10]. It builds knowledge, confidence, skills and the ability to make one’s own informed choices on healthy life-style issues and medical treatment [11]. At the same time, education of health and social care professionals around frailty is important as well because frailty is a relatively new area and many healthcare staff require specific up-skilling [12]. Currently, their education and training are variable and inconsistent across many professions, hindering the provision of high-quality care to older people [9]. Healthcare professionals should remain up to date with developments and best practise in the identification, measurement and management of frailty.

In the UK, the newly published initiative “Frailty: Core Capabilities Framework” (FCCF) [12] outlines in broad terms a set of knowledge and skills for educating frail individuals, their families, carers and healthcare staff with a view to managing frailty more effectively as an LTC; however, it does not describe a specific training course or programme delivery format that can be replicated and applied widely. We therefore carried out a systematic review of known educational programmes to date in order to identify key themes relating to their content and implementation.

## Methods

This systematic review and the protocol were registered with the International Prospective Register of Systematic Reviews (PROSPERO), registration number CRD42019149544 [13]. No other review that covered this question was already in progress. The preferred reporting items for systematic reviews and meta-analyses (PRISMA) guidelines regarding identification, screening, eligibility and inclusion [14] were followed and the final searches were conducted in January 2020. The systematic review was completed as planned with no deviations from the original study protocol.

### Inclusion and exclusion criteria:

The inclusion and exclusion criteria were set according to a standard population, intervention, comparison, outcomes (PICO) domains framework [15]. They encompassed: (1) the population central to the enquiry, i.e. people aged > 65 years with frailty of any severity, their families, carers, and healthcare practitioners; (2) the intervention of interest, i.e. educational programmes aimed at these groups and focusing on frailty, carried out in the community, primary care, secondary care or in care homes; (3) the comparison was any alternative to the intervention if applicable; and (4) the outcomes of interventions, i.e. measurable impact or improvements in clinical indices, frailty status, well-being or quality of life. Reports were excluded if they did not satisfy these characteristics, were not in English language or were published before 2008. The 2008 starting point was because most frailty research and management frameworks occurred afterwards.

### Search strategy:

Computerised databases were searched to identify eligible programmes, studies and other published work relevant to the focus of the systematic review. The National Health Service (NHS) healthcare databases advanced search (HDAS) system [16] provided the platform for the selected databases of EMBASE (Exerpta Medica Database), PubMed, and Ebsco Host. Search terms were selected and combined using standard medical subject headings (MeSH) [17] to identify publications with the desired content. The three aspects of interest were (i) frailty; (ii) education or training; and (iii) study setting. Search terms were used with truncations and the Boolean operators ‘OR’ (horizontal terms) and ‘AND’ (vertical combinations). The search terms were: (i) frail, older people, aged > 65 years, frailty score, mild to moderate frailty, severe frailty, carers, families, relatives, healthcare professionals; (ii) patient education, health literacy, education of patients, self-care, clinical management plans, training programmes, programme evaluation; and (iii) community, primary care, primary healthcare, primary medical care, general practice, family practice, secondary care, hospital care, nursing home, residential home. In addition to the computerised database searches, relevant studies were also sought by performing a manual search of reference lists from retrieved papers, review articles, published conference proceedings, and by discussing with other professionals in the field.

### Screening and selection of publications:

Two reviewers (RV, BP) performed the selection in order to reduce the risk of bias and to increase accuracy. A third reviewer (AF) was also available to discuss any disagreements on what should be included or excluded at the full paper stage. This ensured agreement on inclusion and exclusion of papers, enabling discussion if initially there was a divergent view about any paper. From the initial list of identified publications, any duplicates were removed and the remainder were screened on the basis of the titles and abstracts to select eligible papers. The full-text articles of these were then studied and the final inclusion of studies for qualitative synthesis was agreed.

### Data extraction and quality appraisal:

Data extraction was performed methodically across all papers using a dedicated data extraction form in electronic format that facilitated comparison of the data. The form comprised the following four sections and items: (1) details (authors; title; publication date; type of publication; country of study; funding source); (2) study design (methodology; intervention; inclusion and exclusion criteria; recruitment procedure; study duration; follow-up period); (3) study characteristics (study setting; type of participants; sample size; frailty measurement tool); and (4) frailty outcomes (frailty score; functional state; quality of life; cinical indices; falls; fractures; hospital admissions).

A range of appraisal tools that are commonly used in healthcare research were selected to assess the publications for methodological quality and conduct, risk of bias, validity and usefulness of the results. A single quality appraisal tool would not be suitable for quality appraisal in all the different types of studies. Following statistical advice, four different tools were therefore chosen to cater for the variable study designs. These were: (i) ‘Critical Appraisal Skills Programme (CASP) Qualitative Checklist’ for qualitative studies [18]; (ii) ‘CASP Randomised Controlled Trials Checklist’ for RCTs [19]; (iii) ‘Joanna Briggs Institute Quality Appraisal tool’ [20] for quasi-experimental studies; and (iv) ‘Downs and Black Quality Appraisal tool’ [21] for mixed-methods design and cross-sectional studies. For all papers the quality appraisal tools were applied objectively according to the standard recommendations for their use [22].

### Data analysis and narrative synthesis:

A narrative synthesis was undertaken to bring together the findings from the studies. Popay et al. [23] provide a general framework which was used to guide the narrative synthesis. The framework we used comprised four elements: (1) organising the study findings to describe patterns across the studies and consider how the interventions work and for whom; (2) exploring relationships of study characteristics and findings within and between studies; (3) assessing how widely applicable the findings may be, and (4) assessing robustness of the synthesis.

## Results

**Fig. 1:**
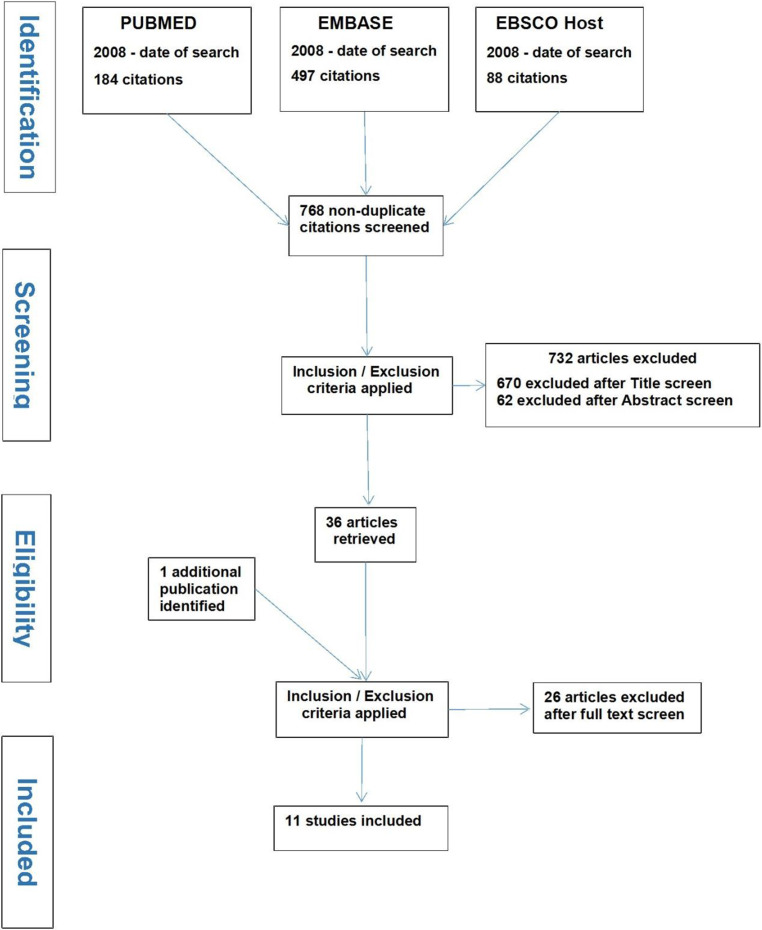
PRISMA flow chart showing the literature search, screening and selection of publications

The computerised database searches yielded 769 papers. They were uploaded to the reference manager software Refworks (Ex Libris, Jerusalem, Israel) which highlighted only one duplicate title. The 768 titles were screened by one reviewer (RV) and reduced to 98 papers that underwent further screening of the abstracts by 2 reviewers (RV and BP). This resulted in 30 papers and 6 conference plenaries. From these, 26 papers were excluded by both reviewers (9 had ineligible participants according to the inclusion criteria; 6 were conference plenaries which were not subsequently published; 5 were systematic reviews that did not aid the research question of this review; 2 were RCT protocols without reported results; 2 were not research papers; 1 opinion piece; 1 could not be accessed). A total of 10 papers were therefore selected. In addition, one other study which did not appear in the computerised searches and involved lay volunteers in the role of educating and training frail people in their homes, met the selection criteria and was also included. The above process is summarised in a PRISMA [24] flow chart in Fig. 1.

A summary of each of the 11 included studies is shown in Table 1. The studies were heterogeneous in design and focus; hence, a meta-analysis was not possible and a narrative synthesis was undertaken. Five (45.5%) of them were undertaken in primary care, five (45.5%) in community settings and one (9%) within secondary care. The study populations were diverse and included frail older people, family members, carers, volunteers, a wide range of healthcare professionals and care home managers. The sample size was also variable, ranging from 12 participants in a qualitative study of semistructured interviews to 603 in a cross-sectional observational study.

**Table 1:** A Summary of the 10 studies included in the systematic review

Study, year, country	Research design and setting	Participants	Objectives	Methodology	Outcomes
*Wallin et al. 2008* [31] Finland	Qualitative secondary care	52 frail older inpatients aged 66–93 years	Assess impact of physiotherapy and health promotion sessions on empowerment of patients and promoting physical activity	Three different exercise groups: (i) highly structured, (ii) guidance plus individualised feedback, (iii) patient-directed with self-regulation	All groups were effective, but each format favoured clients with different needs. A single format is not optimal for all frail older people
*Igareshi et al. 2009* [29] Japan	Quasi-experimental community	150 older people with age-related disorders, mean age 81 years; 32 care managers	Assess efficacy of a preventative care protocol for: (i) maintaining physical independence, balanced diet, falls prevention and social functions of frail people, and (ii) improving skills of care managers	The intervention arm received the structured preventative care. A similar control group received preventive care without use of the protocol	The tool was effective for maintaining self-care strategies. The confidence and practical skills of care managers improved following training to use the care tool
*Chan et al. 2012* [25] Taiwan	RCT Community	117 older people aged 65–79 years	Compare frailty status in 3 similar groups after completing 3 months of different educational and training sessions	Group 1: nutritional plus exercise training; 2: problem-solving therapy; 3: control group. All groups had booklets on diet, exercise and coping strategies	Improvements were found in groups 1 and 2 versus group 3. Group 3 also had improved functional status and quality of life from baseline
*Van der Pol 2018 *[30] Netherlands	Qualitative Community	53 healthcare professionals; 16 older people, mean age 78 years	Develop a teaching framework for health professionals regarding shared decision-making with frail older persons	Subjects identified the required competencies and educational requirements. A consensus was reached for a model of shared decision making	A teaching framework for health professionals was produced to deliver basic knowledge, practical training, and better communication, engagement and collaboration
*Frost et al. 2018* [28] England	Qualitative Community	14 older people aged ≥ 75 years (mild frailty); 12 family carers; 19 community health and social care staff; 8 home care workers	Explore health promotion behaviours and identify key components for new home-based health promotion services	Semi-structured interviews and focus groups with each group of participants	Health promotion services should provide information, enhance motivation, exercise and social networks. Implementation must be individually tailored
*Huang et al. 2019* [33] Taiwan	Cross-sectional Community	603 older people, mean age 71 years	Assess the relationship between health literacy and frailty	The European Health Literacy Survey Questionnaire and the Frailty phenotype criteria were used. Logistic regression identified risk factors for frailty	The extent of physical activity and level of health literacy were associated with frailty status regardless of age and socioeconomic status
*Jadczak et al. 2018 *[26] Australia	Qualitative Community and a Retirement village	12 frail people aged 76–91 years	Explore frail people’s opinions on information about exercise and the role of healthcare personnel in promoting exercise	Semi-structured interviews with community-dwelling people and residents of a retirement village	Retirement village residents had superior advice and information from GPs and other professionals than subjects in the community
*Metzelthin et al. 2013* [27] Netherlands	Mixed methods Primary Care	194 frail people ≥ 75 years; 20 physio- and 6 occupational therapists; 12 GPs; 7 practice nurses	Evaluate a structured inter-disciplinary preventative primary care service delivered by trained staff to prevent disability in older people	A process evaluation using data from logbooks, evaluation forms, semi-structured interviews and focus groups	Many positive aspects about the structure and delivery of the preventive service were reported by frail people and healthcare professionals
*Lally et al. 2018* [32] USA	Qualitative Primary Care	101 nurses; 7 nurse practitioners; 12 social workers; 4 pharmacists; 11 doctors; 15 various trainee health personnel	Develop, implement and evaluate an educational curriculum for training primary care staff to manage frail older patients	Group discussions elicited the key components, e.g. holistic assessment, shared care goal decisions, medicines review, pain management. Staff were then assessed pre- and post-training	Evaluation showed much improved knowledge and confidence of the workforce following the curriculum training sessions
*Willard et al. 2018* [35] Netherlands	Qualitative Community	73 frail people aged > 65 years	Develop and evaluate an interactive, online platform to support independence by providing information, services and stimulating self-care	Frail people’s needs were identified and included in an online platform which was tested for 6 months by the subjects after they received practical training	94% of participants found the platform easy to use; 55% intended to continue using it; and 82% would recommend the platform to others
*Dorner et al.* *2016* [33] Austria	RCT Community	80 frail people aged 65-97 years; 80 lay volunteers	Assess effectiveness of a home-based physical training and nutritional improvement course delivered by volunteers who underwent prior training	A range of physical, nutritional, frailty and quality of life (QOL) parameters were measured in the intervention and control groups	Significant improvements were found in muscle strength, physical performance, nutritional state, frailty status, fear of falling and QOL

The quality appraisal exercise showed that five studies were robust in all [25, 26, 33] or almost all respects [31, 32]. A range of deficiencies or shortcomings were noted in the remaining studies that mostly related to: (i) selection of subjects (e.g. recruitment process not fully described; participants not closely representative of the wider population; subjects not randomised; study groups that were compared had differences) [27–30, 34], (ii) measurement of intervention outcomes (e.g. self-reported outcomes; no blinding of participants and/or assessors) [27–30, 34], (iii) follow-up of subjects (e.g. follow-up not complete in some subjects; characteristics of patients lost to follow-up not given) [29, 34], and (iv) analysis of data (e.g. subjective analysis; possible confounding factors) [27, 30, 34, 35]. Despite these and the possibility of some bias, data from all of the studies added meaningful information that contributed to the objectives of the systematic review.

The exploration and synthesis of the data revealed common findings or similarities in relation to aspects that the studies had targeted or pertaining to the results. These were: accessibility of educational programmes; empowerment; self-care; and health promotion. The various components within each of these themes are described below.

### Accessibility of educational programmes:

The publications recognized that for educational programmes to be successful, accessibility to them is vital and they must also be user-friendly and tailored to the individual preferences and circumstances. The majority of studies employed traditional approaches of various types of face-to-face educational sessions. These included (i) one to one interactions, i.e. consultations [25, 33], interviews [27, 28], discussions [29], counselling [30], (ii) structured group training sessions [25, 28, 31, 32] and (iii) a supply of educational materials in the form of booklets [25, 33] and DVDs [33]. One study explored the application of digital technology [35] and reported that most older people successfully used these technological approaches and had high user satisfaction; however, they noted that ongoing support and engagement were needed in order to achieve sustained use. Also, Lally et al. [32]. provided some web-based resources to supplement their class-based training sessions and they suggested that the whole curriculum they had developed can be translated into telemedicine or video conferencing format. Furthermore, some of the older patients interviewed by Frost et al. [28] in the course of their study had remarked that they would welcome training to use the internet in order to easily access helpful information and advice. All formats of these educational interventions can be used to aid both the prevention and management of frailty.

### Empowerment:

In essence, empowerment is the ability that gives people mastery over their affairs. There is considerable overlap between the studies as regards focusing on empowerment and its elements such as shared decision making [27, 30], care goals setting [31, 32], self-monitoring [31] and the underlying importance of health literacy [34]. Jadczak et al. [26] highlighted the importance of accessibility to educational information. This was supported by Willard et al. [35] recommending provision of reliable information and advice, thus aiding people to feel empowered regarding their health status and to support decision making.

### Self-care:

Self-care was also a common theme across seven studies. This included stimulating, promoting or improving a number of attributes, i.e. motivation [28, 33], problem-solving ability [25], autonomy and independence [28, 31, 35], self-efficacy [27, 33], and the capacity to self-care [29, 35]. There was clear justification for supporting the development of self-care pathways in the prevention and management of frailty. This is seen as a vital element in any educational initiative for achieving improvements in frailty status. Self-care was enhanced in these studies through a range of methods including one to one motivational sessions, counselling and problem-solving.

### Health promotion:

Health promotion encompasses behaviours or actions that have the potential to maintain or enhance physical and mental health and protect against declines. Jadczak et al. [26] showed benefits of actively promoting exercise for the prevention and management of frailty. In support of these findings, Chan et al. [25] found in their RCT that the provision of a combined nutritional and exercise programme resulted in positive, clinically significant effects on frailty status (45% vs 27% of subjects improved by at least one category on the frailty scale, *P* = 0.008); muscle power (2.7 ± 6.1 vs. 0.2 ± 6.7 kg improvement in leg extension power, *P* *=* 0.04); serum vitamin D level (an increase by 4.9 ± 7.7 vs. 1.2 ± 5.4 ng/ml, *P* *=* 0.006); and lower percentage of osteopenia (74% vs. 89%, *P* *=* 0.04) as compared to a problem-solving therapy group or a group not exposed to any intervention. Similarly, the RCT by Dorner et al. [33] found that a home-based implementation of improved nutrition and physical exercises delivered by specifically trained lay individuals resulted in clinically significant improvements in muscle strength (increase by 2.4 kg (95% confidence interval, CI 1.0–3.8), *p* = 0.001), physical performance (increase in Short Physical Performance Battery score by 1.2 (CI 0.3–2.1), *p* = 0.009), nutritional state (increase in Mini Nutritional Assessment-Short Form score by 1.54 (CI 0.51–2.56), *p* = 0.04) and frailty status (reduction in Survey of Health, Ageing and Retirement in Europe Frailty Instrument score by 0.71 (CI ^−^1.07–^−^0.35), *p* < 0.001). The other studies also emphasized the importance of physical activity and exercise [28, 31, 34], balanced diet and nutrition [27, 28, 30, 34], as well as home environmental adjustments and maintaining social activities [28]. Two studies focused on holistic assessment with a view to identifying opportunities for preventive interventions [27, 32].

### Interrelationships of the thematic domains:

The four themes from the studies are clearly interlinked and have a dynamic relationship. Providing frail older individuals access to educational information, guidance and training about frailty improves their understanding and knowledge of the condition [25]. In turn, the greater knowledge empowers them to actively participate in setting their care goals and to gain more ownership of their trajectory through shared decision making [30]. This higher degree of empowerment also enables successful engagement with health professionals in a range of ways to improve their self-efficacy and practical skills that help them to self-care and manage their condition [25, 29, 35]. At the same time, the increased confidence for self-care facilitates lifestyle modifications such as more exercise and healthier diet [25–29, 31, 33].

Benefits for frail individuals can also accrue when their families and carers undergo similar educational processes and can then actively support and assist frail people to achieve more empowerment, self-care, and health promotion [28, 29, 33]. Furthermore, given recent advances in digital technology, there is evidently a place for computer-based and online formats of frailty educational programmes for at least some older individuals, their families, carers and a wide range of healthcare personnel [28, 32, 33, 35].

## Discussion

The key themes that emerged from this systematic review are principally that improving one’s health literacy can empower decision-making in healthcare matters, enhance self-efficacy and practical skills, and enable self-care. Furthermore, increased confidence facilitates life-style modifications such as healthier diet and more exercise. Frail individuals can also benefit when their families and carers undergo similar education and are then able to support frail people in decision-making and maintaining independence. The findings are in step with a report by the World Health Organization emphasising that effective health education is not only about conveying verbal and written information, but also promoting motivation, skills, and confidence so that people can take actions to improve their health [36].

There were some challenges with this systematic review, such as the heterogeneity of research methodology and some issues of suboptimal quality of studies. Also, most of the studies were of qualitative nature and there is an inevitable degree of subjectivity in the process of evaluating them; however, the synthesis followed a structured framework and disciplined approach, and information from all the studies was found to be valuable. There was a fair balance between limitations of various studies and legitimately drawing out the themes that featured prominently, are of general practical relevance widely, and would therefore benefit future research and implementation.

To the best of our knowledge no other reviews have addressed this question, but the key findings enhance the “Frailty: A framework of core capabilities” (FCCF) [12]. This is a recent, authoritative initiative in the UK outlining a package of knowledge and skills (a set of 14 core capabilities) that people with frailty, their families, carers and healthcare professionals should possess in order to improve management of frail older people. It was commissioned by Health Education England following earlier work which recognized that improving support and care for frail older people should be a priority [37]. This systematic review complements the FCCF as the included studies addressed some of the elements of the following FCCF core capabilities: ‘Understanding Frailty’ (capability 1), ‘Frailty Identification and assessment’ (capability 2), ‘Communication’ (capability 4), ‘Families and carers as partners in care’ (capability 5), ‘Preventing and reducing the risk of frailty’ (capability 7), ‘Living well with frailty, promoting independence and community skills’ (capability 8) and ‘Physical and mental health and well-being’ (capability 9). The components of health promotion, empowerment and self-care we elicited also reflect intentions of the FCCF. It is therefore encouraging that much work has taken place to complement the FCCF and that the papers reviewed have demonstrated practical feasibility of a wide range of different approaches together with favourable results of interventions*.* Even though the studies we analysed were carried out before publication of the FCCF, and they were not cited in the FCCF document, they had incorporated into actual practice various elements of what would become the FCCF; however, none of the published studies incorporated a large proportion of the FCCF, each study comprised only individual elements. Hence, there is scope for wider, more comprehensive educational programmes to encompass even more of the FCCF framework.

Unlike the FCCF that does not refer to any specific educational programme methods, the systematic review identified a wide range of educational delivery formats. These included various one-to-one interactions taking place within primary care or in patients’ homes, i.e. consultations, counselling, interviews, and physical assessment and training; group education and training sessions; printed educational material; digital and online platform delivery. All these formats had proved practicable, they were generally well-received and outcomes of interventions were mostly favourable. There was no evidence from these studies that a single format was superior, and clearly all formats have a value to a targeted group. Another important aspect that is likely to have a bearing on effectiveness is the extent to which any educational scheme is individualised or tailor-made to the recipients’ needs. Thus, whenever an educational programme is being formulated, it should stipulate from the outset the type of individuals it is aimed for, and then tailor both the content and delivery format(s) accordingly.

A systematic review undertaken in 2016 analysed structured health education interventions, in 8 comparative clinical trials and 2 case series that aimed to empower nursing home residents and improve their self-management [38]. Educational methods included interactive group sessions, motivational and encouragement strategies, customised counselling based on frail individuals’ needs and preferences, joint goals setting, reasoning exercises, problem-solving training, and printed educational material. Improvements in self-efficacy, self-care behaviours, and activities of daily living were found following the interventions. Whilst frailty per se was not a designated element in that systematic review, evidently it featured prominently as all the subjects were older, resided in care homes, had chronic conditions, disabilities and high dependency level. Half of the studies also included patients with mild dementia. The authors indicated that elements of the structured educational strategies that were key to success of the interventions were individually tailored, interactive, and continually applied interventions. Some of the educational interventions and outcomes from the systematic review by Schoberer et al. [38] evidently overlap with those of our review.

Most of the studies we reviewed were concerned with education and training of frail individuals by a variety of health professionals, and only three studies [28, 29, 33] featured family members, carers or other lay people having a role in educating, supporting frail subjects and enhancing health promotion. A systematic review by Ginis et al. in 2013 reported that education and training delivered by lay individuals can achieve comparable benefits to those that result from interventions by healthcare professionals [39]. In the more recent RCT by Dorner et al. [33], a standardised physical training and nutritional intervention programme for community-dwelling, frail, malnourished subjects was delivered by non-professional individuals who had undergone education and training (4 sessions, each lasting 3 h) covering ageing, frailty, nutrition, exercise and physical training. The frail subjects in the intervention group (received twice weekly, for 12 weeks, home-based physical training exercises, nutritional education and support, and provision of educational material) achieved clinically significant improvements in muscle strength and physical performance [40], nutritional state and frailty status [41], fear of falling [42], QOL and social participation [43]. In this RCT the lay individuals were not relatives or formal carers of the frail subjects, but volunteers who underwent training and then delivered the programme. These findings are very encouraging as they strengthen the view that relatives, carers and others involved in helping and supporting frail older people can be similarly trained to deliver positive outcomes.

The studies we identified that outlined educational programmes for frailty which qualified for review were not many. Also, only two of these studies [27, 29] were an established programme in the wider community, and the rest were exploring the feasibility and effectiveness of new programmes, most of which were evaluated in relatively small groups of subjects. This is in contrast to existing educational programmes for other LTCs such as diabetes and heart failure that are more widely known [44, 45]. Furthermore, generic self-care educational programmes for adults with a range of LTCs and their families have received much interest in recent years [46] and some are well embedded in primary care communities [47] and individual general practice localities [48] in the UK. These programmes have been easily accessible in a range of local venues and have mostly comprised group educational sessions. By way of example, the Walsall Healthcare Self Care Management Programme runs for 2.5 h once a week for 6 weeks [47]. For the present period of COVID-19 epidemic the content of such programmes has been successfully translated into virtual, online courses [47]. These courses aim to help patients and their families understand their role in managing their condition, improve knowledge and skills, make informed decisions about their care, engage in healthy behaviours and to access further support [46, 47]. Clearly, there is appreciable overlap in the principles and themes of content between such generic educational programmes and programmes for frailty. Given the existence of generic programmes that are already funded and staffed, it is conceivable that these can be adjusted to incorporate specific elements relating to frailty education and management as well.

In our systematic review, four of the studies included at least some training of healthcare personnel [27, 29, 30, 32]. Of these publications, two [27, 29] represented an established programme in routine practise and the others were small-scale educational interventions. There does not seem to be many educational programmes that are aimed for a range of healthcare personnel and have potential for widespread uptake. One such resource is the online-based learning package ‘the Frailty Toolkit’ [49] which delivers education and training, a holistic approach to assessment and care, and promotes supported self-care. A face to face version of the Frailty Toolkit training course is also available [50]. Some local health provider organisations have developed their own versions of frailty training in their localities for healthcare personnel involved in the care of frail older people [51]. Clearly, despite the need for standardised educational programmes for health and social care staff, currently there are only few examples in the literature or in practice.

In summary, frailty needs a holistic, interprofessional approach to its identification, assessment and management [52]. This entails a range of multidisciplinary interventions based on older people’s health needs and preferences, care and support planning, medication reviews, physical activity and nutritional optimisation. Promoting effective educational interventions for frailty is a vital component for achieving the delivery of high-quality patient-centered care, and should involve older people who are living with frailty, their families, personal care givers and healthcare professionals who participate in the management of frail individuals. A next step in advancing educational interventions is to translate the themes that emerged from this systematic review, together with the core components of the FCCF, into a widely applicable user-friendly programme that can be evaluated by further research. To maximise its effectiveness, such a programme would need to be comprehensive and tailored to people living with frailty, their families and carers. It should improve knowledge about frailty and its management, motivation, empowerment and self-care skills, health promotion behaviours, home safety, personal support, social network and access to services. To reach the largest possible target audience, the programme content would need to be made available in a range of formats that are easily accessible to individuals with different preferences and circumstances, e.g. face to face consultations and group sessions (at local venues and peoples’ own homes), printed material, and interactive digital and online platforms. As regards the latter format, the current COVID-19 pandemic and social isolation highlight the increasing role of digital and online technologies as a means for many older people and their families to remain in contact and access information, guidance, healthcare services and assistance. Digital and online-based versions of a new educational programme could be formulated to stand alone or as an adjunct to traditional delivery methods.
